# Microbial rewilding in the gut microbiomes of captive ring-tailed lemurs (*Lemur catta*) in Madagascar

**DOI:** 10.1038/s41598-022-26861-0

**Published:** 2022-12-27

**Authors:** Sally L. Bornbusch, Tara A. Clarke, Sylvia Hobilalaina, Honore Soatata Reseva, Marni LaFleur, Christine M. Drea

**Affiliations:** 1 Evolutionary Anthropology Department, Duke University, Durham, NC USA; 2 Center for Conservation Genomics, Smithsonian National Zoological Park and Conservation Biology Institute, Washington, DC USA; 3 Department of Sociology & Anthropology, North Carolina State University, Raleigh, NC USA; 4 Department of Zoology and Animal Biodiversity, University of Antananarivo, Antananarivo, Madagascar; 5 Department of Biological Sciences, University of Toliara, Toliara, Madagascar; 6 Department of Anthropology, University of San Diego, San Diego, CA USA

**Keywords:** Bacteria, Zoology, Conservation biology, Microbial ecology

## Abstract

Microbial rewilding, whereby exposure to naturalistic environments can modulate or augment gut microbiomes and improve host-microbe symbiosis, is being harnessed as an innovative approach to human health, one that may also have significant value to animal care and conservation. To test for microbial rewilding in animal microbiomes, we used a unique population of wild-born ring-tailed lemurs (*Lemur catta*) that were initially held as illegal pets in unnatural settings and, subsequently, relocated to a rescue center in Madagascar where they live in naturalistic environments. Using amplicon and shotgun metagenomic sequencing of lemur and environmental microbiomes, we found multiple lines of evidence for microbial rewilding in lemurs that were transitioned from unnatural to naturalistic environments: A lemur’s duration of exposure to naturalistic settings significantly correlated with (a) increased compositional similarly to the gut communities of wild lemurs, (b) decreased proportions of antibiotic resistance genes that were likely acquired via human contact during pethood, and (c) greater covariation with soil microbiomes from natural habitats. Beyond the inherent psychosocial value of naturalistic environments, we find that actions, such as providing appropriate diets, minimizing contact with humans, and increasing exposure to natural environmental consortia, may assist in maximizing host-microbe symbiosis in animals under human care.

## Introduction

Gut microbiomes (GMBs), critical to animal health^1^, are shaped by various environmental factors, such that altered or unnatural ecosystems (e.g., degraded habitats) have perturbative effects on host-associated communities, with negative health implications for hosts^2,3^. Exposure to key environmental factors has the potential to augment or restore native host-associated microfauna^4^ via an understudied, presumably gradual process known as microbial ‘rewilding.’ The Microbiome Rewilding Hypothesis posits that the restoration of ‘green’ habitats and promotion of diverse environmental microbiomes in urban settings can improve human GMBs and health^5^. If the exposure to or introduction of certain microbial inhabitants can improve host-microbe symbiosis and the host’s ability to adapt to new environments, then rewilding could benefit captive animals transitioning between settings or ecosystems, such as during transfers between captivity facilities, translocations, or reintroductions^6^. Here, we expand the hypothesis to nonhuman primates and test for microbial rewilding in wild-born, captive ring-tailed lemurs (*Lemur catta*) transitioning from highly unnatural settings during illegal pethood to a more natural setting after their surrender to the Lemur Rescue Center (LRC) in Madagascar (Table 1). We ask if, with exposure to naturalistic environments, the GMBs of LRC lemurs better resemble those of pet lemurs or their wild counterparts.

**Table 1:** The study subjects, their habitats, and three factors influencing their gut microbiomes.

Relevant variables	Ring-tailed lemur groups (in chronological order of transitions)		
	Wild	Pet	LRC
Habitat/environment	Natural	Unnatural (townships)	Naturalistic
1. Diet	Native (e.g., wild plants, invertebrates)	Commercial, for humans (e.g., rice, bread, cultivated fruits)	Native forage, supplemented with varied, seasonally available, cultivated fruits and vegetables
2. Direct human contact	None	Constant	Minimal (veterinary and care staff)
3. Environmental exposure	Native microbial communities	Indoor, confined areas in human dwellings	Sheltered, outdoor enclosures with access to natural habitat

Belying traditional dichotomization, both wild and captive settings represent a range of variation known to influence animal GMB structure and function^7^. The GMBs of ring-tailed lemurs, for instance, vary within and between captive and wild settings, such that there is not a universal signal of captivity nor is there a specific, core microbiome that is representative of all of the wild animals^8^ (Supplementary Fig. S1). Here, we focus on three factors known to impact GMB structure and variation across settings: diet, human contact, and exposure to natural environments (Table 1). Notably, the degree of evolutionary mismatch between the diets of wild and captive counterparts is thought to underlie significant variation in GMB diversity and composition^9,10^. In addition, contact with humans can facilitate transmission of microbes and antibiotic resistance genes (ARGs) between humans and other animals^11^. Lastly, exposure to natural environments can mediate the acquisition of environmental microbes and ARGs that can impact host-associated communities and animal health^8,12^. Transitions between settings with different types or degrees of these factors could precipitate changes in multiple aspects of the microbiome, whether via a detrimental perturbation or a beneficial microbial rewilding.

The wild-born lemurs at the LRC have experienced at least two drastic environmental transitions within their lifetime. The first is a perturbative transition when removed from the wild to be kept as pets^13^, and the second is a potentially rewilding transition from pethood to life at the LRC. We use cross-sectional data to first address if time in residency at the LRC correlates with the (a) diversity, (b) phylogenetic composition, and (c) abundance of bacterial taxa in lemur GMBs. We focus on the genera *Bacteroides, Prevotella*, and *Ruminococcus*, as these may serve as biomarkers of host diet type and gut health^14^. Notably, despite the absence of a diverse core GMB among wild and captive ring-tailed lemurs, these microbes are shared and abundant across populations^8^, are also present in the GMBs of other wild and captive primates, and are linked to distinct enterotypes in human GMBs. Investigating variation in these ubiquitous microbes, in combination with broader attributes of microbial communities (e.g., diversity and composition), affords a holistic view of lemur GMB structure, as well as potential insights into changes in functional potential. Next, we also ask if residency at the LRC influences ARG abundance and covariation between lemur GMBs and soil microbiomes from natural habitats. Microbial rewilding in LRC lemurs predicts (i) greater compositional similarity to the GMBs of wild lemurs, (ii) decreased ARG abundance, and (iii) greater covariation with soil microbiomes.

## Methods

### Subjects and samples

The subjects included ring-tailed lemurs living (a) in the wild (n = 139), (b) as pets in Malagasy households (n = 8), and (c) at the LRC in Mangily, Madagascar (n = 25)^8^. Their diets and exposure to humans and environmental microbiomes are summarized in Table 1. Wild lemurs inhabited protected areas (e.g., national parks, community-managed reserves) that varied in habitat type from dry spiny forest to riverine forest. They relied entirely on naturally foraged diets and were constantly exposed to natural environmental microbiomes. Pet lemurs lived in human dwellings in townships located around Toliara, Madagascar. Two of the pet lemurs had limited access to outdoor areas. Their diets were ‘humanized,’ consisting of commercial grains and produce, and they had limited exposure to natural environmental microbiomes. The LRC lemurs were wild-born, former pets that had known dates of surrender to the LRC, where they were socially housed in outdoor enclosures, with access to shelter. They thus could forage freely, obtaining a partial natural diet, supplemented with seasonally available produce, and were exposed to natural environmental microbiomes. Exposure to humans and to ARGs (from combined environmental exposure and/or direct antibiotic administration) was least in the natural populations, maximal in pets, and relatively limited in LRC animals.

We opportunistically collected fresh fecal samples upon observing lemur defecation. To avoid soil contamination of the fecal samples, we removed the outer layer of each fecal pellet. We also collected samples of topsoil (n = 22) from the wild lemurs’ natural habitats, including spiny, dry, and riverine forests in southern Madagascar. When collecting soil, we avoided high-defecation areas (e.g., under sleeping trees) and areas with significant organic matter (e.g., dead vegetation), focusing instead on areas with bare soil, where the lemurs most commonly spent time on the ground. Within these areas, we demarcated a 2–3 m^2^ area and collected topsoil (the top 2–3 cm of soil) from each of five evenly spaced locations. For each area, we pooled the five aliquots of topsoil in a single tube to create a representative soil sample. All fecal and soil samples were preserved in Omnigene.Gut tubes (DNAgenotek, Ontario, Canada)^15^ and, within 8 weeks of collection, were transported to the U.S. and stored at − 80 °C until analysis. No animal handling, manipulation, or experimentation was performed for this study.

### Microbial DNA extraction and sequencing

Following the manufacturer’s protocols for the DNeasy Powersoil kit (QIAGAN, Frederick, MD), we extracted bacterial genomic DNA from fecal and soil samples. We sent aliquots of extracted DNA to Argonne National Laboratory’s Environmental Sequencing facility (Lemont, IL) for library preparation and amplicon sequencing of the V4 region of the 16S rRNA gene. Amplicons were sequenced on a 151 × 151 base pair Illumina MiSeq run^16^.

We sent a subset of the extracted DNA aliquots (wild lemurs, n = 7; pet lemurs, n = 7; LRC lemurs, n = 9) to CosmosID Inc. (Rockville, MD) for shotgun metagenomic sequencing to identify antibiotic resistance genes. DNA libraries were prepared using the Illumina Nextera XT library preparation kit, with a modified protocol^17^. Libraries were then sequenced on an Illumina HiSeq platform 2 × 150 bp. On average, the sequencing yielded approximately 17 million total sequence reads per sample, with an average of 18 million and 10 million reads for fecal and soil samples, respectively. Samples with fewer than 5 million reads (n = 2 samples) were omitted from downstream analyses.

### Bioinformatics and statistical analyses

We processed the 16S rRNA sequence data using a bioinformatics pipeline generated in QIIME2^18,19^. We used the pipeline to join forward and reverse reads, demultiplex, quality filter joined reads and remove chimeras (DADA2 plugin; PHRED scores indicated no quality trimming was needed)^20^, omit non-bacterial sequences (Mitochondria, but not chloroplasts as they can serve as a valuable proxy for diet and environmental exposure^18,21,22^), and generate a phylogenetic tree (mafft program^23^ and fasttree2^24^). To assign taxonomy to our sequence features and generate amplicon sequence variants (ASVs), we de novo trained the Naive Bayes classifier using the SILVA database (ver. 138.1) at 99% sequence similarity^25,26^ and tested the classifier using our representative sequences. After quality filtering, all samples had > 10,000 reads and were retained for downstream analysis. Using QIIME2, we calculated metrics of alpha diversity (Shannon and Faith’s Phylogenetic diversity metric) and beta diversity (weighted and unweighted UniFrac distances) on a rarefied ASV feature table subsampled to 15,000 reads per sample (Supplementary Fig. S2). To examine variation in the abundance of specific microbial taxa, we used R Studio (ver. 4.2.0) to perform a center log-ratio (CLR) transformation on the unrarefied ASV feature table (package ‘compositions’)^27,28^. CLR abundances reflect log-transformed ratios of the raw sequence counts of each taxon over the geometric mean of all other taxa in the sample^29^.

For shotgun metagenomic data, unassembled sequencing reads were directly analyzed using CosmosID’s bioinformatics platform for identifying and profiling ARGs^17,30,31^. The system uses multiple genome databases and a high-performance, data-mining algorithm that disambiguates metagenomic sequence reads. To identify ARGs, we queried the unassembled sequence reads against the CosmosID curated ARG database, which was compiled through assimilation of ARG sequences collected from the published literature, as well as from different open-source databases, including the following: NCBI, CARD, ResFinder, ARDB, ARG-ANNOT, and SEEC. If annotation of a gene conferring resistance was not included in their database, the CosmosID team performed literature searches to determine the class or relevant mechanisms of resistance.

Briefly, and without revealing proprietary information, the CosmosID system uses a high-performance, data-mining k-mer algorithm and highly curated dynamic comparator databases (GenBook®) that rapidly disambiguate millions of short reads into the discrete genomes or genes engendering the particular sequences. The pipeline has two separable comparators: the first consists of a pre-computation phase for reference database and a per-sample computation. The input to the pre-computation phase is a reference microbial genome or antibiotic resistance and virulence gene database, and its output is phylogeny trees, together with sets of variable length k-mer fingerprints (biomarkers) that are uniquely identified with distinct nodes, branches and leaves of the tree. The second per-sample, computational phase searches the hundreds of millions of short sequence reads or contigs from draft assembly against the fingerprint sets. The resulting statistics are analyzed to give fine-grain composition and relative abundance estimates. The second comparator uses edit distance-scoring techniques to compare a target genome or gene with a reference set. The algorithm provides similar functionality to BLAST, but sacrifices some recall precision for a one- or two-order-of-magnitude processing gain. Overall classification precision is maintained through aggregation statistics. Enhanced detection specificity is achieved by running the comparators in sequence. The first comparator finds reads in which there is an exact match with a k-mer uniquely identified with an ARG; the second comparator then statistically scores the entire read against the reference to verify that the read is indeed uniquely identified with that reference. For each sample, the reads from a species are assigned to the strain with the highest aggregation statistics. Outputs include the identity and family, percent gene coverage, and frequency counts of ARGs within each sample. To calculate the proportion of ARGs within a fecal sample, we divided the frequency count of all ARGs or specific gene families by the sample’s total read count.

To calculate covariation between lemur GMBs and soil microbiomes, we used FEAST^32^, a tool that uses fast expectation–maximization, multinomial distributions, and machine-learning classification to model microbial source tracking. FEAST provides “source proportions” of the scaled proportion of each LRC lemur’s GMB community that could be attributed to soil communities from natural habitats or to a default ‘unknown source’ that accounts for microbes not relevant to soil microbiota^32^.

For all LRC lemurs, we calculated time in residency at the LRC as the number of days between surrender date and the date of sample collection (range = 248–2,537 days, standard deviation = 617.7, median = 1,736). Using linear models in R Studio (package ‘stats’), we tested for effects of time in residency at the LRC on lemur GMB diversity, composition, membership, ARGs, and covariation with soil microbiomes. The model included the duration of residency at the LRC as a fixed effect.

### Ethics

Sampling in Madagascar occurred with approval from Madagascar National Parks and appropriate governmental agencies (Ministry of Environment, Ecology, and Forests; permit #s 147/18/MEEF/SG/DGF/DSAP/SCB.Re, 152/19/MEDD/SG/DGEF/DGRNE, 159/16/MEEF/SG/DGF/DSAP/SCB.Re, 154/17/ MEEF/SG/DGF/DSAP/SCB.Re, 156/19/MEEF/SG/DGF/DSAP/SCB.Re). At the time of collection, samples did not require CDC, USDA, or CITES permits. All samples were declared, permits presented, and cleared through U.S. Customs and Border Protection.

## Results

**Figure 1:**
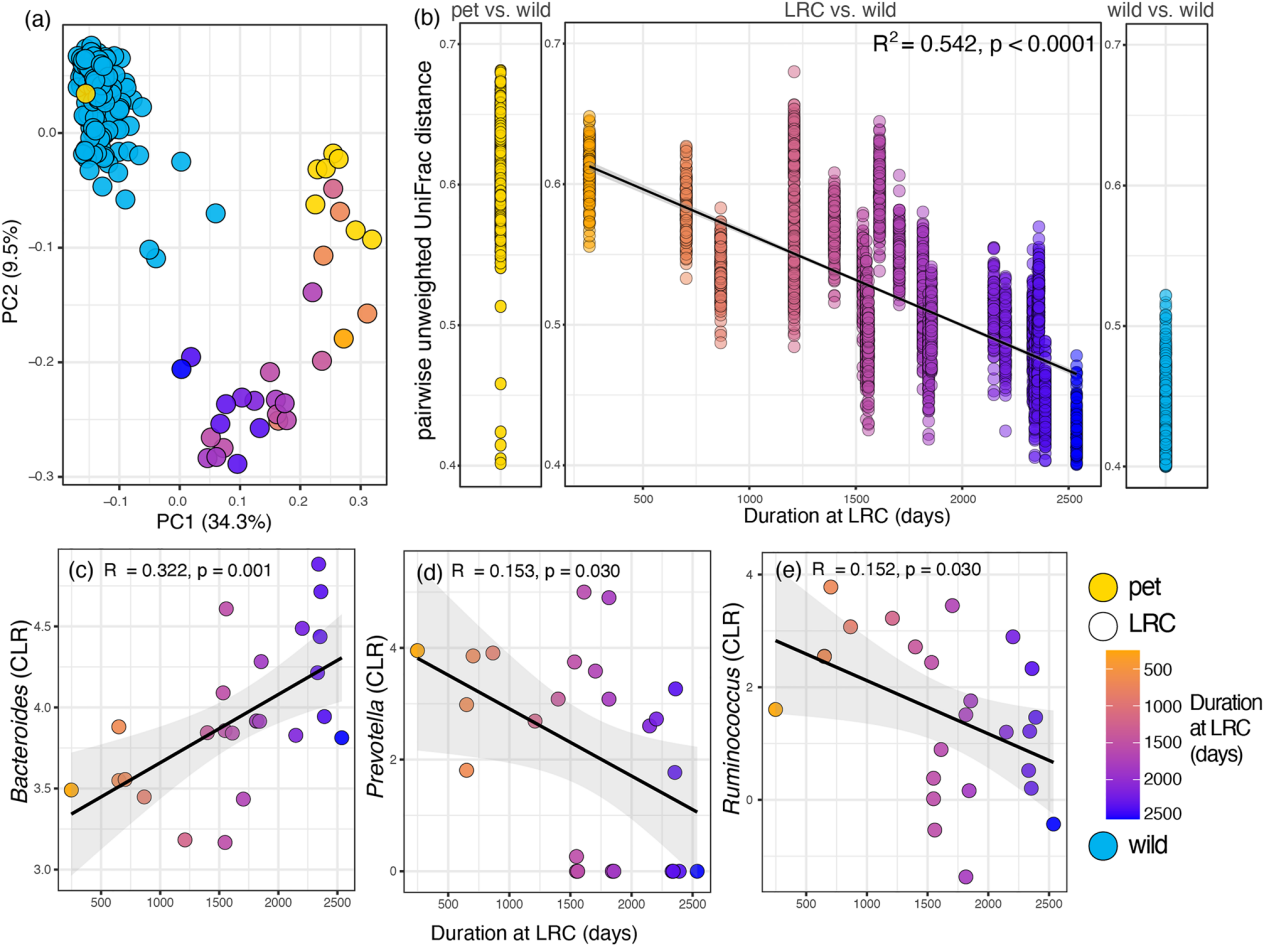
Compositional patterns in the gut microbiomes (GMBs) of three categories of ring-tailed lemurs (*Lemur catta*) in Madagascar. (**a**) ‘Population signatures’ as revealed by principal coordinate plots of unweighted UniFrac distances for wild lemurs (blue), pet lemurs (yellow), and lemurs in semi-natural conditions at the Lemur Rescue Center (LRC; color-graded in relation to duration in residency). (**b**) Rewilding, as revealed by pairwise comparisons, using unweighted UniFrac distance, between the GMBs of pet vs. wild lemurs, LRC vs. wild lemurs, and within wild lemurs. (**c**–**e**) Center log-ratio (CLR)-transformed abundances of Bacteroides, Prevotella, and Ruminococcus in the GMBs of LRC lemurs. Shown are linear trend lines and 95% confidence intervals.; See Table 2 for full statistical results from linear mixed models.

We observed a negative trend in alpha diversity with time in residence at the LRC; nevertheless, the patterns did not reach statistical significance for any metric. In contrast, both compositional measures (or beta diversity) of lemur GMBs significantly correlated with time in residence (Table 2). Specifically, the longer animals resided at the LRC, the more similar their GMB composition was to that of their wild counterparts (Fig. 1a,b; Table 2), consistent with rewilding.

**Table 2:** Results of linear mixed modeling for measures of lemur gut microbiome (a–c) diversity, (d,e) composition, (f–h) center log-ratio (CLR)-transformed abundance of bacterial taxa, (i,j) antibiotic resistance genes, and (k) covariation between lemur and soil microbiomes.

	LRC residency		
	t-value	R-squared	p-value
a. Shannon diversity	− 1.932	0.102	0.065
b. Faith's phylogenetic diversity	− 1.299	0.027	0.207
c. Observed features	− 2.018	0.113	0.055
d. Pairwise unweighted Unifrac distances	**− 64.183**	**0.542**	** < 0.0001**
e. Pairwise weighted Unifrac distances	**− 6.734**	**0.012**	** < 0.0001**
f. *Bacteroides* CLR abundance	**3.526**	**0.322**	**0.001**
g. *Prevotella* CLR abundance	**− 2.313**	**0.153**	**0.030**
h. *Ruminococcus* CLR abundance	**− 2.309**	**0.152**	**0.030**
i. Total ARG relative abundance	**− 4.169**	**0.671**	**0.004**
j. Tetracycline ARG relative abundance	**− 5.330**	**0.774**	**0.001**
k. Source proportion from soil microbiomes	**2.893**	**0.234**	**0.008**

The model included the duration of residency at the Lemur Rescue Center (LRC) as a fixed effect. Significant results are bolded.

The center log-ratio (CLR)-transformed abundance of the *Bacteroides* genus increased significantly with increasing time at the LRC (Fig. 1c). In contrast, the CLR abundances of both the genera *Prevotella* and *Ruminococcus* decreased significantly with increasing time at the LRC (Fig. 1d,e; Table 2).

**Figure 2:**
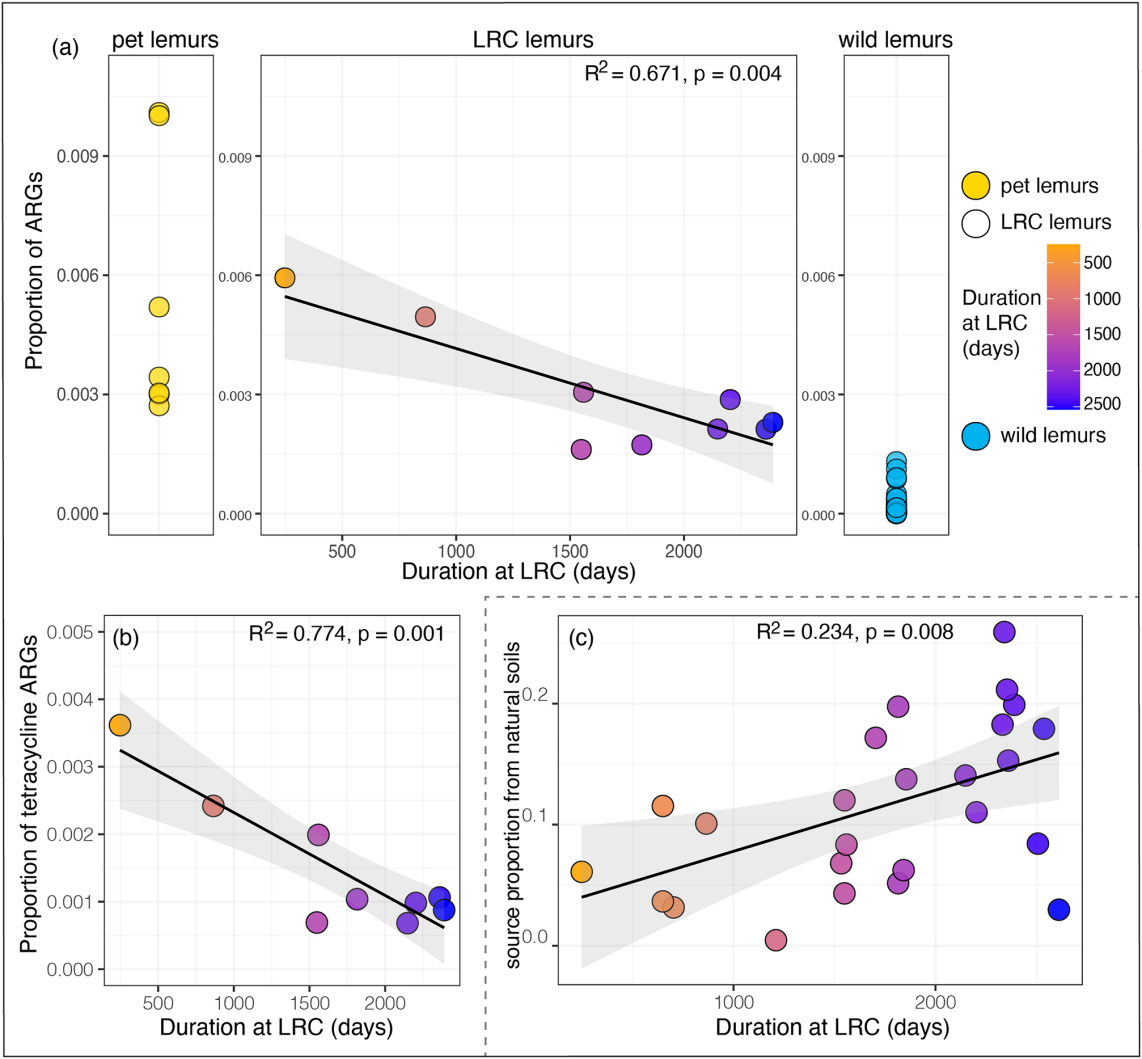
Environmental influences on the gut microbiomes (GMBs) of three categories of ring-tailed lemurs (*Lemur catta*) in Madagascar. Relative abundances of (**a**) total antibiotic resistance genes (ARGs) in wild lemurs (blue), pet lemurs (yellow), and lemurs in semi-natural conditions at the Lemur Rescue Center (LRC; color-graded in relation to duration in residency) and (**b**) tetracycline ARGs in the GMBs of LRC lemurs. (**c**) Total source proportion of soil microbes from natural habitats in the GMBs of LRC lemurs. Shown are linear trend lines and 95% confidence intervals. See Table 2 for full statistical results from linear mixed models.

The total relative abundance of ARGs in the GMBs of LRC lemurs ranged from 0.16–0.59% (mean = 0.29% ± 0.14%). As predicted by rewilding, the relative abundance of total ARGs and of tetracycline ARGs (i.e., the most abundant class of ARGs) decreased significantly with time spent at the LRC (Fig. 2a,b; Table 2).

The source proportion of soil microbes from natural habitats in the GMBs of LRC lemurs—a proxy for covariation between lemur fecal and soil microbiomes – was also significantly and positively correlated with longer residency at the LRC (Fig. 2c; Table 2), again consistent with rewilding.

## Discussion

The present study provides multiple lines of evidence that the Microbiome Rewilding Hypothesis applies not only to humans, but also to wildlife, suggesting that rewilding can serve as a tool to promote animal wellbeing in captivity or during transitional periods, including to ease the microbial reintegration of reintroduced or translocated, endangered species. Notably, for animals that fell victim to the illegal pet trade, but were then relinquished to the LRC, longer periods of exposure to naturalistic environments were strongly linked to more ‘native’ or ‘wild-type’ GMBs, as revealed by microbial community structure, resistance genes, and their covariation with environmental microbiomes. Despite clear patterns in the composition of lemur GMBs, alpha diversity was not significantly correlated with the host’s time spent in naturalistic environments; however, there was a nonsignificant trend for all alpha diversity metrics to decrease with residency at the LRC. Alpha diversity, alone, is increasingly proving to be an inconsistent metric for assessing the influences of environmental factors on host-associated microbiomes and relevant health outcomes^8,33–35^. Although data on animal health would further solidify the relevance of microbial rewilding to animal wellbeing, these results emphasize the importance of incorporating multifaceted microbiome science into animal care and conservation.

Metrics of community composition (i.e., beta diversity) well reflected the predicted and nuanced patterns of environmentally mediated microbial variation^8^. Specifically, longer residency at the LRC was associated with a GMB composition that was more similar to the gut communities of wild lemurs than to those of pet lemurs. The increased similarity was evidenced in both the presence-absence and the abundance-weighted metrics of phylogenetic compositions (i.e., unweighted and weighted UniFrac), indicating that both rare and abundant microbes were driving the pattern of rewilding. We thus explored specific patterns in *Bacteroides*, *Prevotella*, and *Ruminococcus—*three dominant members of primate GMBs^8,36–38^.

*Bacteroides* is a ubiquitous, diverse, and functionally relevant genus in lemur GMBs^35,39^, linked to polysaccharide breakdown and decreased intestinal disease in humans and animal models^40,41^. It is negatively influenced by the common food additives, monosaccharide fructose and glucose^42^. Our evidence of increased *Bacteroides* in the GMBs of LRC lemurs, relative to pet lemurs, could reflect the more appropriate diet provided at the LRC and, in turn, entail decreased disease risk relative to the disease-prone, pet lemurs^43^. Although *Prevotella* has saccharolytic function^44^ similar to Bacteroides, *Prevotella* was significantly decreased in LRC lemurs that had longer residency at the LRC. Both genera rely on similar nutritional resources in the gut, leading to competitive inhibition and contrasting patterns of abundance between the two genera^45^. This competitive relationship has led many to consider abundances of *Prevotella* and *Bacteroides* to be mutually exclusive (i.e., for these genera to be distinct enterotypes), such that the ratio of the two genera may be a proxy for microbial function, host metabolism, and gut health^46,47^. In humans, a lower *Prevotella* to *Bacteroides* ratio—as we see with increased residency at the LRC—has been linked to maintaining or gaining weight when consuming a high-fiber diet^48^. This pattern suggests that the ‘terminal’ microbiomes of LRC lemurs may facilitate or reflect a metabolic shift from malnourishment to improving body condition, achieved by allowing the animals to forage on natural vegetation while being supplemented with the produce-rich LRC diet.

The genus *Ruminococcus*, which was negatively correlated with longer residency at the LRC, is linked to the degradation of resistant dietary starches^49^, including those found in grains, such as rice^50^. Rice is the most widely consumed food in Madagascar and the food most commonly fed to pet lemurs. By contrast, the diets of LRC lemurs do not include rice and are not rich in starch. Importantly, the diets of LRC lemurs include natural forage, which has been shown to dramatically impact GMB diversity and function in folivorous lemurs^51^. Together, the changes in these three dominant taxa—*Bacteroides*, *Prevotella*, and *Ruminococcus*—suggest that the transition from diets associated with pethood to more natural diets at the LRC can facilitate the microbial rewilding process.

Regarding antibiotic resistance, recent studies show that ARG enrichment and propagation can occur in wildlife in the absence of direct clinical treatment with antibiotics^35,52^, namely through the transmission of ARGs between hosts and their social or physical environment^52^. Although pet lemurs in Madagascar almost never receive antibiotics, they have markedly high proportions of ARGs in their GMBs. LRC lemurs, however, are treated with antibiotics in cases of injury or disease. Despite the increased likelihood of LRC lemurs, relative to pets, receiving antibiotic treatment during veterinary care, we found that residency at the LRC, under diminished human contact, significantly correlated with lower proportions of total and tetracycline ARGs. These results suggest a potent role for human contact (or exposure to domesticated animals and their excreta) in ARG transmission to animals, such that minimizing human contact and anthropogenic disturbance would be an important step in the rewilding process.

In terms of the physical environment, beyond acquisition of environmental pathogens^53^, acquisition of commensal or symbiotic microbes is gaining recognition as a component of GMB assembly^54^. The functional relevance of these environmental microbes remains to be determined; yet, there is clear and longstanding evidence that exposure to environmental microbes, or lack thereof, plays a role in shaping animal (including human) immune responses and determining overall health outcomes^5,55–57^. In support of our previous finding that exposure to natural environments dictates environmental acquisition in lemur GMBs^8^, longer residency at the LRC, which equated to greater exposure to naturalistic environments, correlated with greater covariation between lemur GMBs and soil microbiomes from natural habitats. In addition to the inherent psychological and behavioral value of providing naturalistic environments for wildlife under human care, we find that exposure to rich, natural microbial landscapes has the potential to augment host-associated communities.

Together, our results suggest that microbial rewilding is a multi-faceted and gradual process that includes host-associated and environmental microbial communities. Moreover, we suggest that providing appropriate diets, minimizing contact with humans, and increasing exposure to natural environmental consortia are actionable steps that can promote microbial rewilding in captive animals. These actions may be particularly valuable for animals slated to undergo environmental transitions or reintroduction^6,58^. By rewilding host GMBs prior to the transition, we may be able to prime animals for success in their new environments. Going forward, the collection of longitudinal data on the GMBs and overall health of animals undergoing environmental transitions will be essential for understanding the microbial dynamics that drive microbial rewilding and their ultimate relevance to the animal host.

## Supplementary Information

Supplementary Figures.

## Data Availability

The 16S sequencing reads are available in the National Center for Biotechnology Information's Sequence Read Archive (BioProject ID #PRJNA821395). Data on antibiotic resistance genes are deposited in the Open Science Framework repository, link: https://osf.io/vkr2f/, https://doi.org/10.17605/OSF.IO/VKR2F. The full metagenomic library is available upon reasonable request from the corresponding author (SLB).
