# Supplementary material for: Microbial rewilding in the gut microbiomes of captive ring-tailed lemurs (Lemur catta) in Madagascar
Source: Sci Rep. 2022 Dec 27;12:22388. doi: 10.1038/s41598-022-26861-0 (PMC9794702; doi:10.1038/s41598-022-26861-0)
Supplement: Supplementary file 1 — Supplementary Figures. [file 41598_2022_26861_MOESM1_ESM.docx]

**Supplementary Information**

*Microbial rewilding in the gut microbiomes of captive ring-tailed lemurs (Lemur catta) in Madagascar*

**Supplementary Figure S1**


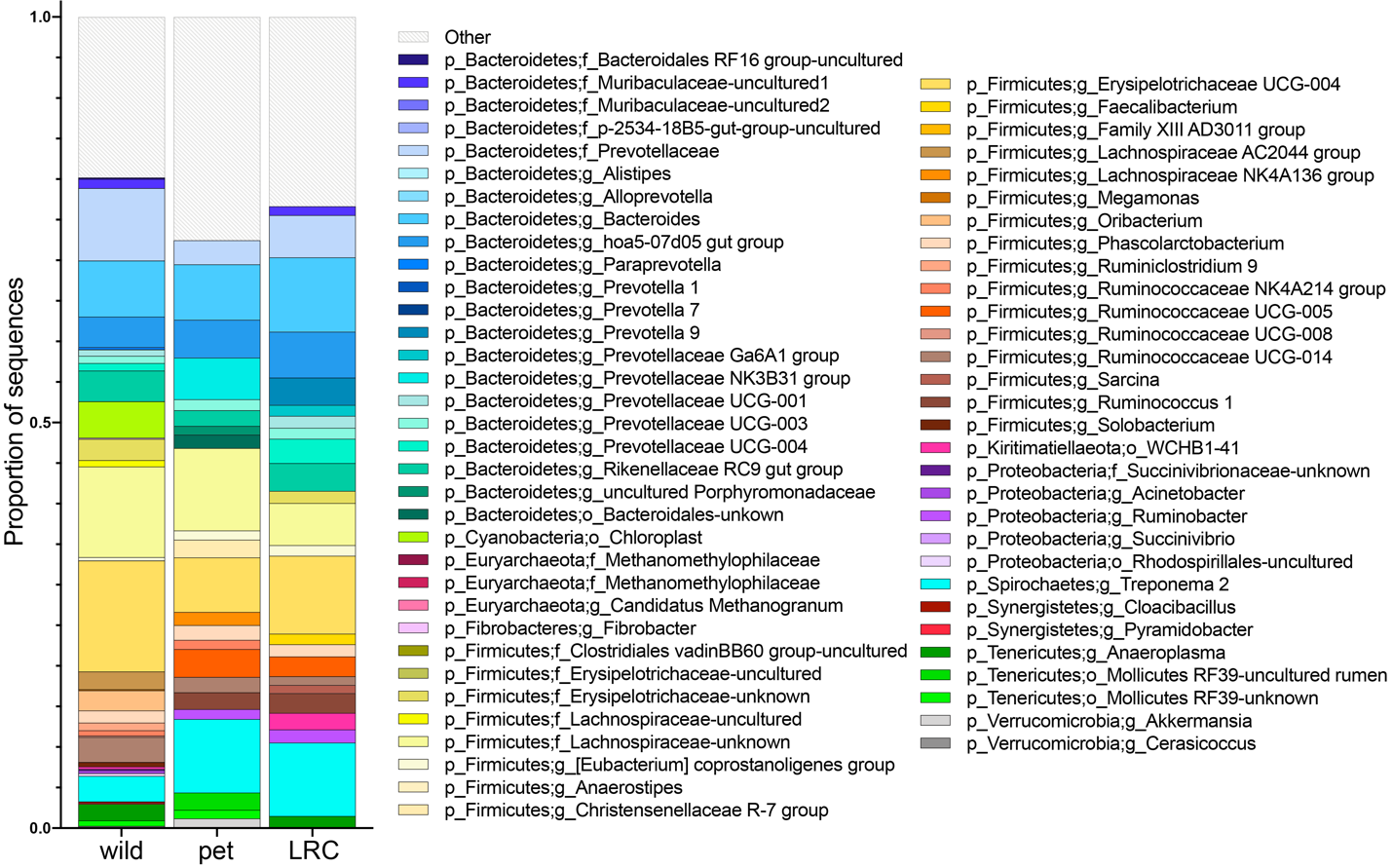


Figure S1. Mean proportion of sequences assigned to microbial taxa across wild, pet, and rescued ring-tailed lemurs (at the Lemur Rescue Center or LRC). Taxa are identified by phylum and deepest possible taxonomic level (i.e., genus level or above); those representing < 1% of the microbiomes were combined into the category “Other.” (Adapted from Bornbusch et al., 2021)

Bornbusch SL, Greene LK, Rahobilalaina S, Calkins S, Rothman RS, Clarke TA, LaFleur M, Drea CM. Gut microbiota of ring-tailed lemurs (*Lemur catta*) vary across natural and captive populations and correlate with environmental microbiota. *Anim Microbiome* 2022; **4**: 1–19.

**Supplementary Figure S2**


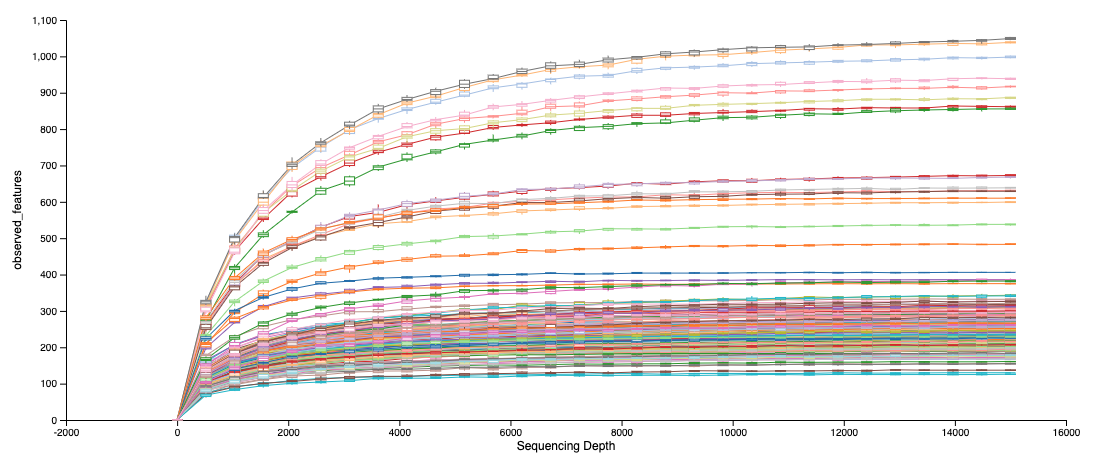


Figure S2. Plot of observed features (i.e., the number of unique amplicon sequence variants) rarefied to a sequencing depth of 15,000 sequence reads for all samples.
